# Familial aggregation and heritability of hypertension in Han population in Shanghai China: a case-control study

**DOI:** 10.1186/s40885-019-0122-z

**Published:** 2019-08-15

**Authors:** An-le Li, Xiang Fang, Yi-ying Zhang, Qian Peng, Xian-hong Yin

**Affiliations:** Jiading District Center for Disease Control and Prevention, Shanghai, China

**Keywords:** Hypertension, Heritability, Segregation ratio, Familial aggregation

## Abstract

**Background:**

To explore the familial aggregation and heritability of hypertension in Han in Shanghai China.

**Methods:**

According to l:l matched pairs design, 342 patients of hypertension and 342 controls were selected and investigate their nuclear family members in the case-control study. The method of genetic epidemiology research was used to explore the familial aggregation and heritability of hypertension.

**Results:**

The prevalence rate of hypertension of first-degree relatives was significantly higher (34.44%) than that of second- degree relatives (17.60%) and third-degree relatives (13.51%) in Han Population in Shanghai China. Separation ratio p was 0.217, and prevalence rate of case group relatives was higher than that of control group relatives. The results showed a phenomenon of familial aggregation in the distribution of hypertension. The heritability of first- degree relatives was 49.51%; that of second-degree relatives and third-degree relatives were respectively 23.42 and 21.41%.

**Conclusion:**

The distribution of essential hypertension has phenomenon of familial aggregation in Han Population in Shanghai China. The separation ratio of essential hypertension in this study shows that essential hypertension conform to the characteristics of multigene genetic disease. The heritability of first-degree relatives is bigger than that of second-degree relatives and third-degree relatives.

## Introduction

Essential hypertension is a disease caused by a variety of factors. Some studies suggest that there are two major factors affecting the incidence of hypertension: one is the genetic factor; the other is environmental factors, such as overweight and obesity, smoking, mental stress, lack of physical activity, high salt intake, and excessive drinking, anxiety, irritability and other emotional factors, lead to an increased serum epinephrine and blood pressure [1–10]; Shortage of sleep has been demonstrated to be associated with elevated blood pressure as well [11].

Essential hypertension (EH) is a complex disease influenced by genetic and environmental factors, in addition to genetic factors, several evidence suggest that stress contributes to the genesis of EH. There are many studies on genes or chromosomal loci that are linked to EH, but the results are controversial [12–19]. This difference is probably due to the small sample size of single study and the different source of the population. According to the current research data at home and abroad, it is recognized that hypertension is caused by both environmental and genetic factors, the influence of genetic factors on hypertension is about 20–55% [20, 21].

It is now believed that the genetic factor of hypertension is made up of a number of pathogenic genes that have a small but cumulative effect. But so far, the susceptibility genes for hypertension have not been finalized. Because of the different origin and stratification of the population, it is a challenge to find the gene of essential hypertension. The main reason for the challenge may be genetic heterogeneity and confounding factors. The genes associated with essential hypertension may be a group and micro effect mode, each of which plays a different role in different stages of the formation and development of hypertension and interacts with each other. The interaction between genes and genes, genes and environment, environment and environment leads to different risk of hypertension among different populations and individuals. By the dose effect relationship of quantitative characters, the critical threshold of the occurrence of the disease is reached, and the genetic susceptibility to essential hypertension is determined simultaneously.

This study uses methods of case-control and genetic epidemiology study, based on the investigation of the essential hypertension patient family and the control family, to explore the familial aggregation and heritability of hypertension in Shanghai China.

## Methods

### Determination of the core family in case group

All patients were randomly selected from hypertension registry and follow-up management system in Jiading district in Shanghai. The diagnosis of case was defined by the outpatient or inpatient diagnosis in clinical hospitals, the blood pressure was based on clinical blood pressure in hospitals. All patients were confirmed by hospital and verified by follow-up in the community, and they were able to correctly respond to the investigators for health information of themselves and their nuclear family members. Every patient gave informed consent to participate in the study which was approved by the local ethics committee (JD-2016-KY-18). Total 342 patients were selected, among these patients there are Han races, and there are 173 male cases, average age 61.3 ± 11.3 years old; 169 female cases, average age 63.6 ± 10.1 years old.

### Determination of the core family in control group

According to l:l matched pairs design, all controls population had no hypertension, and controls are required the same sex as cases, same race, living in the same community, and the difference of age is not more than 5 years old and at the same age group. Every control gave informed consent to participate in the study. They were able to correctly respond to the investigators for the health information of themselves and their nuclear family members. Total 342 control population were selected, among these controls there are Han races, and there are 173 male, average age 61.1 ± 11.1 years old; 169 female, average age 62.4 ± 10.3 years old.

### Standard of case diagnosis and inclusion

All patient patients were over 18 years old, who has been diagnosed by the hospital as essential hypertension, excluding secondary hypertension, such as renal artery stenosis, pheochromocytoma, hyperaldosteronism and other diseases.

### Family survey method and content

Investigation was conducted by trained public health investigators, using a unified questionnaire. Using direct survey method, the contents of the questionnaire mainly include: age, sex, race, age of onset, diagnosis time, hospital name, family members and so on. The family investigation mainly includes: spouses, parents, siblings, children, uncle, aunt and cousin. The criteria for judging whether all the respondents had essential hypertension (all relatives of the case and the control population): whether they had been diagnosed with essential hypertension in the hospital before this investigation. If they had been diagnosed with essential hypertension in the hospital, it is “Yes”; if they had not been diagnosed, it is “No”.

### Statistical analysis method

Statistical analyses were performed using the statistical software package (IBM SPSS statistics version 21). When *P* values < 0.05, the difference is considered statistically significant. Using Falconer method to calculate heritability of three degree relatives, and the genetic model was evaluated by modified Weinberg method [22].

#### Separation ratio estimation

Separation ratio P is: P = $\frac{\boldsymbol{R}-\boldsymbol{J}}{\boldsymbol{T}-\boldsymbol{J}}$ (T is the total number of siblings; R is the number of case in their siblings; J is family number only one case among their siblings).

Standard error (SE) is: SE(p) $=\sqrt{\frac{\left(R-J\right)\left(T-R\right)}{{\left(T-J\right)}^2}+\frac{2Q{\left(T-R\right)}^2}{{\left(T-J\right)}^4}}$ (Q is siblings number of two patient, and the others are the same as above).

#### Heritability estimation

Heritability (h^2^) is: h^2^ = $\frac{\mathbf{1}}{\boldsymbol{r}}\ast \frac{\boldsymbol{P}\mathbf{0}\left(\boldsymbol{X}\mathbf{0}-\boldsymbol{X}\mathbf{1}\right)}{\boldsymbol{a}\mathbf{0}}$ (r is genetic similarity coefficient; p_0_ is non-incidence probability of relatives in the control group, p_0_ = 1-q_0_, q_0_ is the incidence of relatives in control group); x_0_ is normal deviation of the average of relatives susceptibility to threshold in the control group; x_1_ is normal deviation of the average of relatives susceptibility to threshold in the case group; a_0_ is normal deviation of patient’s susceptibility to susceptibility of relatives in the control group. x_0_, x_1_ and a_0_ value can obtained by referring to the simplified Falconer table according to the prevalence rate).

Standard error (SE) is: SE(h^2^) = $\frac{1}{r}\ast \sqrt{\frac{P1}{A{(a1)}^2}\ast {\left(\frac{P0}{a0}\right)}^2}$ (p_1_ is non-incidence probability of relatives in the case group, p_1_ = 1-q_1_, q_1_ is the incidence of relatives in the case group; A is case number; a_1_ is normal deviation of patient’s susceptibility to susceptibility of relatives in the case group; and the others are the same as above. a_1_and a_0_ value can obtained by referring to the simplified Falconer table according to the prevalence rate).

## Results

### Basic situation of the family

In this study, 342 patient cases and 342 control population were investigated, average age in the case group was 62.4 ± 10.7 years old, and average age in the control group was 61.7 ± 10.7 years old. Among these investigated people, they are all Han races, so no racial analysis will be conducted. The difference of age between case group and control group were not significant (t_age_ = 0.894, P_age_ = 0.372). The comparison of investigation variables between case group and control group showed in Table 1.

**Table 1:** The comparison of investigation variables between case group and control group

		Control group	Case group	x	*p*
Sex	male	175	171	0.119	0.730
	female	167	171		
Education	Illiteracy	20	22	6.567	0.161
	Primary School	84	83		
	Junior High	133	154		
	High School	69	67		
	College/above	34	18		
Blood type	unknown	135	126	3.523	0.620
	A	50	58		
	B	49	55		
	O	65	72		
	AB	41	33		
Character	unknown	3	9	4.252	0.373
	introversion	143	143		
	partial introversion	100	104		
	extroversion	46	48		
	partial extroversion	49	39		
Work pressure	not	217	222	3.08	0.545
	little	96	86		
	big	23	24		
	very big	4	8		
Occupation	physical	154	152		
	mental	90	71	12.197	0.016
	freelance	26	17		
	no work	70	104		
Sleep time	inadequate	43	35		
	sometimes adequate	30	50	6.998	0.072
	adequate	268	259		
Exercise	Never	63	65		
	Occasionally	116	130	1.454	0.483
	Often	162	148		
Drink	Never	236	251		
	Occasionally	65	59	2.068	0.558
	Often	38	34		
Smoking	never	225	219		
	quit	22	40	6.544	0.088
	occasionally	20	24		
	always	72	61		
Diet	salty	65	88	5.738	0.057
	light	104	110		
	balanced	172	146		

Total number of nuclear family members investigated was 8599 (4353 in case group, 4246 in control group), and the average nuclear family member was 12.57 (case group is 12.73; control group is 12.42). Among the investigated population, there were 3386 in the first-degree relatives, the average nuclear family member was 4.95; and 3103 in the second- degree relatives, the average nuclear family member was 4.54; and 2110 in the third-degree relatives, the average nuclear family member was 3.08.

### Family prevalence

There were 1997 hypertensive patient has been clearly diagnosed in the hospital among this investigated population in this study, the average prevalence was 23.22%. The prevalence rate of hypertension of first-degree relatives was 34.44%; prevalence rate of second- degree relatives was 17.60%; prevalence rate of third-degree relatives was 13.51%. This result showed that the order of hypertension prevalence rate was as follows: first-degree relatives > second- degree relatives > third-degree relatives.

All prevalence rates of hypertension of case group relatives were significantly higher than that of control group relatives. The result showed a phenomenon of familial aggregation in the distribution of hypertension.

Among these family members and relatives in case and control group, there were 517 hypertension in parent population, the prevalence was 44.84%; 549 in sibling population, the prevalence was 39.96%; 100 in offspring population, the average prevalence was 11.64%; 291 in paternal siblings population (brother and sister of father), the prevalence was 18.27%; 255 in maternal siblings population (brother and sister of mother), the prevalence was 16.89%; 46 in paternal cousin population, the prevalence was 13.11%; 239 in maternal cousin population, the prevalence was 13.59. The difference of hypertension prevalence in three level relative populations between case group and control group were significant (see Table 2).

**Table 2:** The hypertension prevalence rate (%) of relatives in case and control group

	Control group			Case group			x^2^	*p*
	total	disease	prevalence	total	disease	prevalence		
First-degree relatives								
Parent	570	203	35.61	583	314	53.86	14.853	0.000
Siblings	704	217	30.82	670	332	49.55	21.557	0.000
Offspring	440	33	7.50	419	67	15.99	11.899	0.001
	1714	453	26.43	1672	713	42.64	48.167	0.000
Second- degree relatives								
Paternal siblings	791	123	15.55	802	168	20.95	5.375	0.020
Maternal siblings	740	107	14.46	770	148	19.22	4.339	0.037
	1531	230	15.02	1572	316	20.10	9.635	0.002
Third-degree relatives								
Paternal cousin	176	14	7.95	175	32	18.29	6.330	0.012
Maternal cousin	825	97	11.76	934	142	15.20	3.897	0.046
	1001	111	11.09	1109	174	15.69	7.282	0.007
Total	4246	794	18.70	4353	1203	27.64	83.649	0.000

### Separation ratio analysis

According Table 3 and separation ratio calculated formula above, separation ratio p = (891–580)/(2013–580) = 0.217; standard error = SQRT (0.0001186 + 2*163* 2.98E-07) =0.015, and 95% confidence interval (CI) was (0.188, 0.245). According to the theory of genetic epidemiology, theoretical separation ratio is 50% in monogenic dominant inheritance; theoretical separation ratio is 25% in monogenic recessive inheritance. This result suggested that the genetic pattern of hypertension does not belong to monogenic dominant or recessive hereditary disease. This study is inclined to consider essential hypertension may be multigene recessive hereditary disease.

**Table 3:** The separation ratio estimate in first-degree relatives

Siblings number per household	Household number	Total siblings number(T)	Cases among siblings (R)	Household number have only one case among siblings (J)
1	167	167	167	167
2	163	326	154	129
3	119	357	160	87
4	106	424	168	89
5	75	375	121	43
6	29	174	53	18
7	17	119	49	14
8	2	16	6	1
9	5	45	11	3
10	1	10	2	0
Total	684	2013	891	580

### Heritability analysis

According to methods of genetic epidemiology analysis, genetic similarity coefficient of first, second and third degree relatives were respectively 0.5, 0.25 and 0.125. the calculated results showed that the heritability of hypertension of different degree relatives was different. In this study, the heritability of first-degree relatives was 49.508%; and heritability of second-degree relatives and third-degree relatives were respectively 23.416 and 21.407% (see Table 4). The order of heritability was as follows: first-degree relatives > second- degree relatives > third-degree relatives.

**Table 4:** The heritability (%) and 95% CI of hypertension of different degree relatives

	Prevalence	x	a	h^2^	SE	95%CI
First-degree relatives						
Control group	26.43	0.681	1.278	49.508%	0.0004	(49.507, 49.508%)
Case group	42.64	0.251	0.962			
Second-degree relatives						
Control group	15.02	1.062	1.575	23.416%	0.0003	(23.415, 23.416%)
Case group	20.10	0.845	1.406			
Third-degree relatives						
Control group	11.09	1.301	1.761	21.407%	0.0003	(21.407, 21.408%)
Case group	15.69	1.089	1.623			

## Discussion

It is well known that the genetic factors play an important basic role in the occurrence of hypertension, and family history is a sign reflecting the main role of genetic factors. It’s showed that family history plays an important role in the development of hypertension through interaction with acquired risk factors such as body mass index (BMI), and the interaction of family history and BMI is greater than the sum of two independent actions [23]. In this study, the genetic epidemiological results showed that the prevalence of hypertension in three degree relatives was different. The prevalence in the first degree relatives (including parents, siblings and offspring) was the highest (34.44%), the prevalence in the second degree relatives (including paternal siblings and maternal siblings) was the next (17.60%), and the prevalence in the third degree relatives (including paternal cousin and maternal cousin) was lowest (13.51%). The difference of hypertension prevalence among three degree relatives in Han in Shanghai China were significantly (*P* < 0.05). It was suggested that genetic factors have obvious influence on the occurrence of essential hypertension, and the genetic potency may be decrease with the increase of degree relatives.

In this study, Hypertension confirmation was based on the definite diagnosis of the hospital. Considering incomplete recognition of hypertension patients in this investigated population, the separation ratio calculation was used the modified calculation formula. With regard to the genetic pattern of essential hypertension, the result in this study showed the separation ratio of essential hypertension was 0.217, 95% CI was (0.188, 0.245). This result showed that separation ratio of essential hypertension was lower than 0.25 of the genetic rule of Mendel single gene, which conforms to the characteristics of multigene genetic disease [24, 25]. It indicated that the occurrence of essential hypertension is a common effect produced by multiple genes with minor effects under certain environmental factors, rather than the action of single gene. The effect of a single gene may be relatively small.

At present, method of estimating the heritability of polygenic disease is generally accepted to calculate the heritability based on the threshold theory of Falconer [26]. In this study, the heritability of first-degree relatives was 49.508%, within the average heritability reported in the relevant literature (20–55%) [20, 21], and lower than Tibetan (77.2%) [27]. it indicates that there are also ethnic differences in hypertension susceptibility genes among different ethnic groups. In this study, the heritability of second-degree relatives and third-degree relatives were respectively 23.42 and 21.41%. The order of hypertension heritability was as follows: first-degree relatives > second- degree relatives > third-degree relatives. From the above stratified analysis results, the effect of genetic factors on the incidence of essential hypertension may be more obvious in first-degree relatives, and fell obviously as the level of kinship declined. The above results show that the occurrence of hypertension in the Han population in Shanghai, China, is affected by both genetic and environmental factors, which is consistent with the reports of relevant manufacturers [28–30]. The formation of polygenic diseases is influenced by both the genetic and environmental factors, in which the size of the genetic factor is represented by the heritability. The estimated heritability in this study could not completely eliminate the confounding effect of environmental factors. We will further analyze the interaction between genetic factors and environmental factors in the next study.

## Conclusion

The distribution of essential hypertension has phenomenon of familial aggregation in Han Population in Shanghai China.

The separation ratio of essential hypertension in this study shows that essential hypertension conform to the characteristics of multigene genetic disease.

The heritability of first-degree relatives is bigger than that of second-degree relatives and third-degree relatives.

## Data Availability

The questionnaire and database supporting the conclusions of this article are available, through contact with anle_li@aliyun.com.
